# Expectations Shaped Elsewhere: Refugee Experiences of Healthcare in Aotearoa New Zealand

**DOI:** 10.1111/hex.70492

**Published:** 2026-05-20

**Authors:** Molly George, Rebekah Kennedy, Lauralie Richard, Pauline Norris

**Affiliations:** ^1^ Social Anthropology Programme University of Otago Dunedin Otago New Zealand; ^2^ Va′a o Tautai – Centre for Pacific Health University of Otago Dunedin Otago New Zealand; ^3^ General Practice and Rural Health University of Otago Dunedin Otago New Zealand

**Keywords:** expectations, GPs, healthcare, New Zealand, primary care, refugees

## Abstract

**Background:**

We know that many refugees arrive in host countries, including Aotearoa New Zealand, with complex health needs and are at risk of poorer outcomes. Little attention has been paid to the expectations and understandings of care that they bring with them and how these shape their health experiences. Many refugees come from hospital‐based healthcare systems with direct, timely access to specialists, tests and procedures but resettle into primary care systems where GPs act as gatekeepers to public and private specialists and nonemergency hospital services.

**Design/Methods:**

Refugee health literature often focuses on provider perspectives. With two recent projects, we explored refugees' experiences of healthcare and access to medicines in southern New Zealand. The first gathered refugees' experiences of healthcare through 20 open‐ended interviews. The second project followed five refugee households over a year through in‐person interviews, video calls and texts. Both generated extensive qualitative data, analysed and coded thematically by research teams.

**Results and Discussion:**

Both studies found that refugees felt significant dissatisfaction with healthcare in New Zealand. This stems in large part because refugees' expectations of healthcare are shaped by previous experiences of healthcare overseas. New to a primary care system, refugees described it as strange and slow and were openly frustrated by its more limited access to tests, referrals and follow‐up—features that previously signified care. Different prescribing practices (namely, more frugal, with a perceived over‐reliance on painkillers) also sowed scepticism of GPs. Waitlists deepened doubts about the system's ability to care for them.

**Conclusion:**

Different experiences and understandings of care, formed overseas, contribute significantly to refugees' dissatisfaction with healthcare in New Zealand. Listening to their perspectives and understanding how and why their expectations are not being met is an important step in improving refugee healthcare provision and outcomes.

**PPIE:**

Our methodological approach prioritised relationship‐building and responsiveness; we spent significant time building relationships with service providers who work with refugees and interpreters before beginning. Service providers helped develop our research and interview questions and facilitated recruitment. As early themes emerged during interviews with refugees, they were integrated into subsequent interviews for participants to verify or refute. In the first study, we brought early findings to community partners and two participating households for validation. One household continued into the second project. The longitudinal approach of the second study fostered sustained participant engagement. Both studies centred the underrepresented voices of refugees themselves, aiming to bring their perspectives to practitioners and, in doing so, contribute to improving refugee healthcare experiences.

## 1 Introduction

### 1.1 Healthcare Is Falling Short of Former Refugees' Needs and Expectations

It is widely acknowledged that many refugees arrive in host countries, including Aotearoa New Zealand, with complex healthcare needs and are at risk of poorer health outcomes [1, 2, 3, 4]. However, far less attention has been paid to the expectations and understandings of care that they bring with them [5] and how these shape their health outcomes and experiences. The most reported point of access to healthcare for migrant populations, including refugees, is the primary healthcare network of their country of resettlement [1]. This is a very different system than many refugees have previously experienced: hospital‐based systems with direct and timely access to specialists and relatively quick access to tests and procedures [3, 6]. Access to healthcare, then, is a common challenge for migrant and refugee populations, often due to difficulties navigating a primary care system [7]. Furthermore, patient satisfaction is consistently reported to be lower in primary care systems compared to specialist care systems [1, 8]. The gap in understanding how refugees' prior experiences shape their navigation of primary care systems risks perpetuating poor health outcomes and inequities from the outset of their resettlement.

Much of the existing literature focuses on the provider perspective—namely, general practitioners (GPs)—acknowledging challenges they face [9, 10, 11]. This is appropriate considering that GPs are often the first point of contact for refugees within primary care systems, and they face significant challenges in providing care for complex social and health needs within a limited, overburdened system not designed to support refugee populations [1, 10, 12]. However, the lack of refugees' input and perspective in these studies is a major limitation.

With two recent projects, we set out to explore refugees' experiences of healthcare and access to medicines in southern New Zealand. Both studies found clear evidence of former refugees' dissatisfaction with aspects of New Zealand′s primary care system, as well as practical barriers to accessing healthcare services and medicines. This article draws on qualitative data generated through the two projects and focuses on one of the primary findings across both: refugees' experiences of healthcare *before* arrival in New Zealand (in their homelands and transition countries) shape their expectations and experiences of healthcare *in* New Zealand. In many cases, these pre‐existing expectations contribute to the dissatisfaction refugees experience with New Zealand's healthcare system. Although healthcare delivery does vary across New Zealand, our conversations with providers (during our research, dissemination and the creation of resources for general practices) indicate that the experiences described here are shared by many refugees and the clinicians who care for them nationwide. The findings are therefore relevant not only to New Zealand but to primary care systems internationally that care for refugees who are accustomed to hospital‐based care with more direct access to specialists.

### 1.2 Context

In New Zealand, the refugee quota program allows up to 1500 refugees to be resettled annually (‘quota refugees’ in the UNHRQ system). When refugees arrive in New Zealand, they enter a residential orientation programme called Te Āhuru Mōwai o Aotearoa—Mangere Refugee Resettlement Centre [13]. The Centre operates as a primary care practice for newly arrived refugees for the 5 weeks they reside there, before moving to their permanent resettlement sites in regions of the country [14]. They receive medical screening and treatment, and upon leaving, their records should transfer with them to a GP. They are considered residents and have the same entitlements as all New Zealanders to subsidised primary healthcare [13].

New Zealand's health system is largely publicly funded through taxation [15]. As in the United Kingdom, the Netherlands, Sweden, Australia, Canada and many other countries, GPs act as gatekeepers to public and private specialists and nonemergency hospital services [16]. GPs are doctors who have completed specialty training in general practice and are fellows of the Royal New Zealand College of General Practice [17]. They work largely in private practices. These practices increasingly also employ other health professionals like nurse practitioners [15], pharmacists [18] and health improvement practitioners (who deliver mental health and behavioural support) [19]. Practices provide a range of services from health promotion and prevention to treatment and management of acute and chronic conditions [1, 7].

Charges for GP visits typically vary from around $20 (approx. £9) to around $80 (approx. £36) [20]. User charges are a significant barrier to access for many people [20]. Most prescription medicines are free for those with low incomes or those aged under 14 or over 64. There is a $5 (approximately £2.50) charge per item for everyone else, until they reach an annual threshold of 20 items [21]. In common with many other countries, there are waiting lists for seeing a specialist and for hospital treatment [22]. Despite universal coverage, there are significant inequities in access to and outcomes from healthcare in New Zealand [15]. Ongoing workforce shortages, particularly of GPs, exacerbate these [23].

## 2 Methods

Research involving resettled refugees has methodological and ethical complexities [1, 24, 25]. Our approach prioritised relationship‐building and responsiveness to both refugee service providers and refugee participants. Our process began with L.R. (author 3) forming relationships, over several months, with service providers who work with refugees (i.e., healthcare navigators, primary health organisations [PHOs], English language schools, Red Cross and other organisations). The first author, M.G., was brought into these relationships and, through them, learned crucial information about engaging with local refugee communities appropriately. Through these community partners, M.G. learned how crucial the ‘right’ interpreters would be (e.g., do not use interpreters from the same country as the participants for one particular group of refugees). She was connected to the local interpreters most trusted by refugees. Before beginning fieldwork, M.G. spent time building relationships with three interpreters. All of these service providers helped develop our research and interview questions; for example, adding questions about nutrition and women's health. These service providers also helped recruit participants for each study by spreading the word for us, distributing our project information sheets and in some cases making first contact with potential participants on our behalf. We made sure we had diversity in household composition, ethnicity, age and gender.

M.G. is an experienced qualitative interviewer with expertise working with many marginalised communities. Participant safety and comfort were prioritised by providing information about the research, the researchers and the interpreters in advance and again upon meeting. M.G. always offered participants a choice of where to meet (all chose their own homes in the first study and most of the second study) and arrived with a small plate of food to share. M.G. readily adapted to participants' preferences around recording, written/verbal consent, gender‐related protocols and more. In both studies, the participants led the direction of the interviews. As early themes emerged, they were integrated into subsequent conversations for participants to verify or refute.

**The first project**, ‘Refugees' Journeys to and Through Healthcare in New Zealand’ (led by L.R.), gathered first‐hand accounts of refugees' experiences of New Zealand's healthcare system in the Southern region. M.G., with a trusted interpreter, conducted 20 open‐ended interviews with 31 former refugees from four countries. A detailed methodological discussion of this project, including our emphasis on relationship‐building, recruitment, rapport and participant safety, can be found elsewhere [24]. Interviews lasted approximately 2 h and were audio‐recorded. M.G. recorded extensive fieldnotes in spoken and written form after each interview. The interviews were conducted in English with an interpreter, and the English language portions were transcribed alongside the fieldnotes before beginning thematic analysis [26].

Our analysis process was inductive, with themes emerging from repeated close engagement with the data, rather than through applying pre‐existing hypotheses or frameworks [26]. To begin, all research team members examined the transcripts and fieldnotes, noting and discussing early, emergent trends. Following these discussions, M.G. began coding the transcripts and fieldnotes with ATLAS.ti, and brought the developing codes back to the team for further discussion. Through successive rounds of reading, coding and collective interpretation, the team validated codes and continued to identify broader recurring themes. These themes included, for example, ‘Care as something needing to be done’, ‘Primary care as strange, slow, limiting’ and ‘Wait times equating to lack of care’. We brought early, anonymised findings—in the form of our emergent themes—to our initial community partners (service providers working with refugees) and to two participating households for member checking and validation.

**In the second project**, ‘Access to Medicines: Exploring Lived Experience to Inform Policies and Programs’ (led by P.N.), four researchers followed 21 households (5 refugee, 5 Pacific, 5 Māori and 6 Pākehā living in poverty) and their experiences of seeking healthcare and medicines for a year. Recruitment of the five refugee households drew on the connections made before and during the first project. One refugee household from the first project joined the second. Others were recruited with the help of healthcare navigators and interpreters who had come to know and trust us. M.G. maintained contact with the participating refugee households for over a year through in‐home interviews, WhatsApp text and voice messages, and during times of high Covid alerts, through video calls (all facilitated by our interpreters). Fieldnotes and the English language portions of interviews were transcribed. We maintained a strong priority on the voices of the refugees themselves, seeking to understand participants' accounts of healthcare experiences within the broader context of their lives. The longitudinal study design demonstrates sustained engagement and trust developed with participants and gave more insight into the compounding and ongoing struggles refugee households faced when accessing healthcare and medicines [27].

As with the first project, our analytical approach was inductive, but our process was slightly different. Given the large volume of material generated in this more longitudinal study, M.G. initially broadly identified any material related to healthcare or medicines using NVivo. The four researchers then met in person for several days to examine this material collectively, discussing and agreeing by consensus on broad, inductively derived themes related to experiences of healthcare and access to medicines. We then reread the relevant material and coded it to these themes. This process was iterative; we moved between the transcripts, fieldnotes and the developing themes, refining our themes while staying grounded in the refugees' accounts of their healthcare experiences. Some of the themes that emerged include, for example: ‘Medicines as suboptimal treatment’ and ‘Communication (or lack of) in pharmacies’. See Table 1 for a brief summary of the two projects.

**Table 1:** Summary of the two studies.

	Study 1: Refugees' journeys to and through healthcare in New Zealand	Study 2: Access to medicines: Exploring lived experience to inform policies and programs
Participants	20 refugee households (31 participants) from 4 countries. Diversity in household composition, ages and gender.	5 refugee households from 3 countries. Diversity in household composition, ages and gender.
Recruitment	Recruited with help from trusted service providers.	One household participated in Study 1. Others recruited with help from trusted interpreters and healthcare navigators.
Data collection methods	In‐person interviews (approx. 2 h each). Follow‐up messages with some participants.	Sustained engagement over a year via in‐person interviews, video calls, text and voice messages.
	Copious fieldnotes.	Copious fieldnotes.
Interpretation	Facilitated by a pre‐identified interpreter. Follow‐up messages translated by same interpreter.	Facilitated by a pre‐identified interpreter. Follow‐up messages translated by same interpreter.
Data recording	All interviews recorded except one where notes were taken. Interviews and fieldnotes transcribed.	All spoken interactions, and all fieldnotes, recorded and transcribed.
Duration of study	Single interviews, with some follow‐up with a few participants.	Longitudinal: Followed each household for over a year with some form of contact every 3–5 weeks.
Data analysis	Inductive analysis—Themes emerged from repeated, close engagement with data rather than from pre‐existing frameworks.	Inductive analysis—Themes emerged from repeated, close engagement with data rather than from pre‐existing frameworks.
	Researchers all read transcripts and fieldnotes, identifying and discussing early emergent trends.	Due to extremely large data set, M.G. first used NVivo to broadly identify all material related to experiences of healthcare and medicines.
	M.G. coded transcripts and fieldnotes using ATLAS.ti, bringing developing codes back to team for successive discussion and refinement.	Research team met in person, over several days, to review this material—Discussing and identifying broad, inductively derived themes. We then coded the material in detail to these themes, refining them while staying grounded in refugees' accounts.
	Findings were shared with community partners and 2 participating households for feedback and validation.	

During analysis for the second project, we identified a consistent theme that appeared strongly across both studies: refugees seem to assess their experience of healthcare and medicines in New Zealand *in comparison to* previous healthcare experiences overseas. By this stage, all the involved researchers were intimately familiar with the original data sets and the themes that had emerged from them. We also brought a student researcher (R.K.) on board, and M.G. and R.K. worked together to further check the data in both studies that pertained to refugees' comparisons of healthcare experiences. Again, they worked inductively allowing themes to emerge from the refugees' own accounts of healthcare experiences both in New Zealand and abroad. This process confirmed many of our earlier findings from the two projects and arranged them in this new overarching theme of overseas experiences of healthcare shaping expectations and experiences within New Zealand. M.G. has taken these results back to some the original community partners (healthcare navigators) and to some GPs for feedback and verification. The themes identified in this collated body of data are the results presented in this article. All names used are pseudonyms, and identifying characteristics (including countries of origin) have been changed or omitted to protect anonymity. Both studies were approved by the University of Otago ethics committee.

While refugee participants were not involved as formal research collaborators, our approach was grounded in sustained engagement and responsiveness to the community and participants. The goal in both studies was to centre and amplify the voices of refugees themselves. Our aim now is to bring these insights to the healthcare practitioners who care for them. In this way, the research prioritises refugee patient voices to shape and improve healthcare experiences for refugees.

## 3 Results

### 3.1 GP System: New, Slow and Strange

Our participants' experiences of New Zealand healthcare were shaped, in large part, by their experiences of other healthcare systems in their homelands and transition countries. Most notably, previous healthcare experience was largely of hospital‐based systems and included timely direct access to specialists: ‘Back home they have medical centres similar to the GP centres, but it's different in the sense that here it's mostly all of them are general practitioners. But back home they have lots of doctors, lots of different specialities. And so, they assess for example what's wrong with you… [and] you go to a specialist straight away. You'll get what you want from the specialist’ (Leila).

Having to see a GP in New Zealand, instead of or before a specialist, was completely novel for participants: ‘This is new for us because it was not like this in [home country]… That was shocking, that we can't go directly to see a specialist!’ (Abdul). Having to go through a GP was strange, even perceived as futile: ‘Oh my God, I can't believe it… Wait ‐ if I need someone to look after my ear, why would I go to a GP? I usually go to an ENT’ (Yasmin).

#### 3.1.1 Fewer Tests, Referrals, No ‘Physical’ Exam and So Forth

Participants frequently described GPs offering far fewer physical examinations, tests, referrals, medications and follow‐up than they were accustomed to overseas. These services *constituted* healthcare in their previous experience, so the absence of them was experienced as *lack of care*. Several participants found the lack of physical examination disconcerting, describing GP appointments as largely or entirely conversation‐based. Mona, for example, went to her GP with some inflammation, but the GP only asked her to describe the problem; she did not look and feel her body. Back in her homeland, Mona said, if she got any kind of inflammation, the doctor would ‘check her properly’ with a physical exam and then give injections, tablets or something for the bath. Mona left the appointment feeling she had not been given professional care. The inflammation remained, but seemingly more important, her *anxiety* about the inflammation remained because it had not been investigated through physical touch and further testing. Yasmin echoed this sentiment about touch, explaining that she had gotten into the habit of asking the GP to ‘please touch’ whatever part of her body she was concerned about. She found it odd how ‘GPs in NZ just look at you… You have to touch me to know what's happening… Never send me to a doctor that won't touch and examine me’. New Zealand's healthcare was experienced as ‘hands off’, which was perceived as lacking thoroughness and professionalism.

Participants expressed frustration at often being told, in various ways, that ‘everything is normal’ when expressing a concern. Ali described going to the GP with a particular ache and was told it was a part of getting old; the GP even said that she herself experienced the same thing. Similarly, Lucia described going to the GP, describing her problem and being met with a look of: ‘And??’ This look, Lucia felt, was dismissive and insulting. Zahra and several others described being told ‘there was no problem’ because blood tests were normal. For many participants, this felt dismissive and like an unprofessional lack of investigation and/or follow‐up. Mina described going to the GP repeatedly with the same concern until some medication was suggested and further testing was ordered. Only then did Mina feel she was being ‘taken seriously’ and care was finally being given. Some described changing GPs to one who would order additional testing.

#### 3.1.2 Wait Lists—They Exacerbate Everything Above

Even when a GP did order tests, procedures or referrals to specialists, the GP does not have the power to bring these next steps to fruition in New Zealand's overburdened, under‐resourced system. Participants reported waiting, with increasing unease and frustration, for the *actual healthcare* (which were perceived to be the tests, the physical examination, the referral or specialist appointment) to begin. Describing being waitlisted for a procedure on his eye, another participant explained he was almost ‘one hundred percent certain’ that in his homeland they would have done something immediately. The participant hastily added that in his homeland almost everything ‘has turned bad’. But, he said, they would have done something for his eye quickly. Another participant said something similar: ‘It's not that it's all so super‐fast in [homeland], but at least if you go there and you're already quite concerned about something, they immediately refer you to someone else. Comparatively speaking, things happen faster there than they happen here… if you need a surgeon then you get a surgeon right away. But not here’. Andre felt the same way when describing a problem that his wife has with her eyes. They were told, ‘we cannot do anything about it until it is worse’. He questioned, ‘they will wait for her to become blind?’ with exasperation at the seemingly uncaring system and providers.

Importantly, participants described these delays (several days to see a GP and weeks, months or even years for a test, procedure or specialist consultation) as not just a surprising systemic problem but *as a complete absence of care*. Furthermore, this perceived lack of care felt personal. For example, Abdul described having made several appointments, each time complaining of back pain but always being told ‘only: “wait.”′ He elaborated, ‘When I have pain I have pain, I want someone to consider that I am in pain. If they want to refer me to see a specialist, I want to see the specialist in three days, in one week, in two weeks and not several months. When I go to see a doctor and I am in pain, I feel the pain; they don't feel it, they don't see it, they don't touch it, they don't understand it… I want them to consider that I am in pain’. Negative stories and anxiety about waitlists circulated. Even hearing stories of waitlists left some with anxiety and lack of confidence that the system will care for them when they need it: ‘I'm worried, if I have pain, I'll have to wait a year’.

#### 3.1.3 Different Prescribing Practices

Refugees described different prescribing practices in New Zealand, which they had mixed feelings about. Sometimes, they felt GPs prescribed too many medicines as a way of ‘fobbing off’ the patients instead of providing ‘real care’. Specifically, a perceived over‐reliance on paracetamol was described repeatedly and reportedly had become a joke amongst refugees. Isabella said: paracetamol is ‘the solution to everything here’; another participant laughed that she had so much paracetamol she could supply it to others, saving them a trip to the GP—where they would inevitably just be given paracetamol. Ali described the care plan in New Zealand as taking pain pills until you become utterly desperate. Sayed and others said healthcare in New Zealand effectively amounts to ‘take Panadol [paracetamol] and wait’. Noor expressed the futility of painkillers, in her experience: ‘The medications I am getting from here, I have got a big box full of medications, but they are not useful. They are all painkillers’.

At other times, participants described GP prescribing practices as haphazard, a sort of ‘trial and error’ approach. This added to the scepticism of GPs because it was assumed specialists would have more specific diagnoses, treatments and medicines. As one participant said: ‘Because [GPs] don't know exactly what's going on, they might prescribe you with medications, instead of providing or recommending the actual treatment’. Another participant described feeling like a guinea pig with the GP just trialling a series of different medicines rather than making a referral to a specialist (who would, the participant assumed, know the right medication or have better methods for determining the best medicine). In the instances described above, medicines were experienced as suboptimal treatment.

On the other hand, sometimes participants felt they were not provided with *enough* medicine, especially medicines they were used to receiving overseas, which was sometimes even described as excessive. One participant laughingly described needing a suitcase for a doctor's appointment in his homeland, just to carry the medications the doctor would prescribe. In contrast, in New Zealand, he had seen just one tablet being given to a patient. Another participant described being given pain medicine in two or five tablets at a time for reasons she did not understand. One participant dealt with chronic health problems and was continuously frustrated that the medicines prescribed overseas were not prescribed readily, or at all, by New Zealand doctors: ‘the GP doesn't prescribe it’. Others described a similar experience of asking for medicines they received overseas, but being denied those medicines by a New Zealand practitioner.

Refugees also described some medications, such as antibiotics, being available over‐the‐counter in their previous countries of residence, but controlled through prescription in New Zealand—often by doctors who were far more reluctant to prescribe them: ‘antibiotics are like gold here’. Many participants lacked clarity about the different ways of accessing medicines in New Zealand, for example, which are over‐the‐counter versus prescription, which are subsidised versus not subsidised. New Zealand's comparatively frugal prescribing practices and more prescription‐only access to some medicines were confusing and frustrating.

### 3.2 Distrust and Lack of Confidence in This System to Care for Them

The above findings demonstrate that the New Zealand healthcare system was a surprising disappointment for many refugees. Several participants relayed their assumption, before arrival, that New Zealand's healthcare system would be *better* than that of their transition countries and troubled homelands. One participant incredulously described her inability to ‘reconcile the fact that [New Zealand] is supposed to be a more advanced country’ yet healthcare was, in her perception, inferior.

Some participants made general statements of distrust and disappointment, like ‘I don't trust the system’ and ‘I'm just not convinced about the whole system’. Others described a feeling of having been outright lied to. This was often around waitlists, as it was for Martina, who described having been repeatedly told that a needed procedure would be done in 3 months. A year had passed. ‘Perhaps three months just sounds better than one year’, she said, suggesting that she might be deliberately given inaccurate information to placate her. Another participant muttered a similar sentiment referring to her healthcare in New Zealand: ‘we've just been told so many lies’. Two participants noted less frequent routine testing as reason for doubting New Zealand's healthcare system. For example, both Ana and Ariana were accustomed to annual pap smears, whereas in New Zealand, at the time of the interviews, this screening was usually every 2 years. Ana worries about the repercussions of delayed diagnoses and feels the system does not have her best interest at heart. Ana's family has created a ‘Plan B: to learn English very well, then get work, earn money, save a lot, and then when we have enough, if we get sick, we will go to another country and have treatments there and not here’.

This lack of confidence in the system has resulted in many participants simply not seeking care anymore. Jamila's ‘2 year anniversary’ of arrival in the country was approaching, and after this point, she would need to pay for GP appointments. With money so tight, she said she would rather ‘drink water and take Panadol’ than go to the GP because, she said, this would be the unsatisfying outcome anyway. Ali says he, and some others he knows, have gone back to herbs and traditional medicines, instead. Haya was perhaps the most outspoken about not bothering to seek care anymore. She has significant episodes of terrible pain, but no longer calls the doctor about it. Her son pushes her to go to the hospital when the episodes occur, but she refuses: ‘they are not going to do anything anyway’.

## 4 Discussion

When migrants and refugees move to another country, they take with them their prior experiences, beliefs and practices of health and healthcare [28], but only a relatively small amount of literature has looked at how these previous experiences and understandings of healthcare translate to a new system in a host country. Many refugees come from hospital‐based systems with direct access to specialists and timely access to tests and some procedures. This differs considerably from the primary care system they arrive to in New Zealand and most other resettlement countries [1, 3, 6].

New Zealand's primary care system, like others, utilises GPs as a first step or entry point to healthcare. GPs act as ‘gatekeepers’, and there are a variety of rationales for this. These include managing demand on the hospital system, controlling costs [8], prioritising the use of hospital services, reducing supplier‐induced demand by specialists [16], promoting co‐ordination of care, responding to shortages of specialists, reducing waiting times and reducing adverse effects from overtreatment [16]. Aoki et al. [29] argue that gatekeeping aims to ensure appropriate use of healthcare services and reduce possible adverse effects of unnecessary care [29]. In France, the introduction of a gate‐keeping system led to a reduction in specialist visits [30]. However, some studies have also shown adverse effects from gatekeeping: it may lead to a reduction in cancer survival (potentially through delayed treatment) [16] and lower patient satisfaction [8].

Refugees coming from hospital‐based systems may have expectations of having needs resolved (through seeing a specialist, undergoing testing, getting a diagnosis and a prescription or a follow‐up plan of some sort) all in 1 day or in quick succession [5]. We propose that for refugees, dissatisfaction with New Zealand's healthcare system stems in part from the novel encounter of these GP ‘gatekeepers’. Like us, Lawrence and Kearns [31] found that refugees in New Zealand were unfamiliar with processes of referral, waitlists and the requirement of appointments to see a GP. But it is more than unfamiliarity; at times, GPs are perceived to be ‘blockades’ to the kind of care refugees are used to. This resonates with Patel et al.'s [5] finding that some refugees in Australia perceived the *GP him or herself to be delaying progress* towards diagnosis, treatment or resolution. O'Donnell et al. [6] found that refugees unfamiliar with the primary care system before arriving in the United Kingdom lacked confidence in GPs, feeling that they may not have specialist knowledge.

Not only does primary care involve an additional entry step, and is inherently slower, our participants felt that GPs ordered far fewer tests, procedures and referrals than what they were used to. Patel et al. [5] have called these actions ‘visible care’ or ‘visible markers of care’ and found that in the absence of visible markers of care, there was significant dissatisfaction. We too found that many refugees in New Zealand would define care as these ‘visible actions’. This understanding of care is based on their previous experiences of frequent testing, interventions, heavy prescribing practices and more. These visible actions *constituted* care in the past; without them, care is simply not occurring. This lack of visible ‘markers of care’ is experienced as lack of care at all.

Looking at this through a medical anthropological lens, ‘care’ is enacted through symbols, and when carer and patient apply shared meaning to a symbol of care (in this case, tests, imaging, referrals and ‘visual care’), the patient and caregiver are joined in this shared understanding [32]. But when these taken‐for‐granted symbols of care are different between patient and carer, there is a ‘fracture between community members' expectations and the care that they receive’ [5, p. 644]. This idea is supported by the fact that our participants did not always describe their relationships with a GP negatively. Refugees mentioned ‘pleasant’ GP visits or noted how nice it was to have one, single point of contact who ‘knew their story’ more intimately. Several participants described their GPs as warm and caring and appreciated the oversight of a good GP: ‘It is good to have a GP, a doctor who knows the family and the history… and they have all the records’. However, nearly all participants, including those who spoke well of their GPs, described leaving GP appointments feeling unsatisfied because even these good interactions did not necessarily constitute *healthcare*.

Interestingly, while much attention has been paid to the challenges of language barriers, translation and interpretation in refugee healthcare, Patel et al. [5] were careful to point out that this difference in expectations of care is not solved by sharing a language (‘language concordant care’) when the healthcare provider's understanding of ‘good care’ has been shaped by significant part by being trained or working within a primary care system. Language barriers do not fully explain the lack of access to or refugees' dissatisfaction with healthcare in a primary care settlement country. The challenge may instead be about the poor translation of expectations of care and understandings of what constitutes effective and valid treatment [28, 31].

Building on this, we found that when healthcare in the resettlement country does not meet refugees' expectations, dissatisfaction and distrust can emerge, potentially leading, in some cases, to delayed presentation or avoidance of care. As one participant told us, sometimes advice circulates around the refugee community about seeking healthcare: ‘Don't bother’. Dissatisfaction, then comes full circle, becoming a barrier to care. If the experience of seeking care leads to negative feelings, this might prevent refugees from seeking future care, which could contribute to growing health inequities [3].

New Zealand prescribing practices and medicines also differed significantly from what refugee participants expected. In low‐ and middle‐income countries, effective pharmaceutical regulation may be lacking. For example, in Iran, there is a lack of transparency and accountability in, amongst other things, licensing of pharmaceutical companies, drug registration, procurement and distribution. There are no written rules about pharmaceutical advertising to doctors (although advertising to the public is forbidden) [33]. In some low‐ and middle‐income countries, pharmaceutical companies directly financially reward doctors for prescribing their medicines [34, 35], providing an incentive for doctors to prescribe large numbers of medicines. In addition, the availability of ‘prescription‐only′ medicines without prescription is common in many countries [36, 37, 38]. This increases the risks of inappropriate treatment and/or adverse effects. In the case of antibiotics in particular, there are concerns about the development and spread of antibiotic resistance [39, 40].

These overseas contexts—of more prescribing and over‐the‐counter access to medicines—could go some way to explaining refugees' perceptions that prescribing practices are frugal in New Zealand. For some refugees, the more restrictive prescribing practices are frustrating and suggest a lack of care. Lawrence and Kearns [31] found that some refugees in Auckland, New Zealand felt like a healthcare appointment that does not result in a prescription is ‘not good value’. If medicines are a major marker of care for refugees based on previous experience, the inaccessibility of them in New Zealand is then seen as a lack of care that further feeds distrust. But *which* medicines are prescribed matters. Like us, other research has found that refugees complained about over‐reliance on painkillers [2, 7].

Chronic pain is highly prevalent amongst refugees [41], and some of our participants had high expectations that GPs would prescribe medicines that would successfully treat their pain. One participant, for example, recalled getting injections for her chronic pain in her transition country, an option that was not offered in New Zealand. Refugees such as these are frustrated about only being prescribed paracetamol and experience it as being ‘fobbed off’ and not cared for. This is echoed in O'Donnell et al.'s [6] finding that refugees in the United Kingdom perceived some prescribed medications as ‘a replacement for serious professional attention’ [6, p. 174]. This exacerbated the scepticism of GPs and further harmed trust in the primary care system. But the distrust and frustration go further than just doubting ‘the system’. Sayed explained that the perceived poor quality of medicines and prescribing practices in New Zealand meant that his health blocked him from participating in New Zealand life: ‘I want to go to school. I want to learn English. I want to be able to stand on my own feet and have an independent life but I can't’. If they could get ‘the right’ medicines—which they often felt that had been taking before coming to New Zealand—it could change the quality of their life and settlement in New Zealand.

The high physical and mental health needs of refugees are well documented and, importantly, are known to stem not just from pre‐existing conditions and trauma, but also the challenges of resettlement itself once in the host country. Resettlement issues that affect health are known to include discrimination, unemployment, social isolation and more [31]. We add to this that another important issue impeding successful resettlement is dissatisfaction with healthcare. Our research sits within a small body of literature that closely examines the expectations of healthcare that refugees come with when resettling in a new country. We further emphasise that these differences between expectations of care (based on direct‐access systems) and the reality of care in the settlement country (in a primary care system with significant limitations) are experienced as a *lack of care* altogether. In New Zealand, many refugees feel the healthcare system they have entered consists of GP appointments where nothing seems to happen, long waitlists and paracetamol. The impact of this cannot be overstated. One refugee told us, ‘Back home I was traumatized by violence. Here I'm traumatized by health’.

Richard et al. [10] found that primary carers in New Zealand feel a duty and moral commitment to refugee care and often go above and beyond. These carers are faced with managing refugees' sometimes unrealistic expectations of healthcare delivery within New Zealand's system [10, 31]. Robertshaw et al. [12] found that differing base understandings of health makes care provision challenging. Our work confirms that different experiences and understandings of care are a major contributing factor to refugees' dissatisfaction with care in New Zealand. We shed light on refugees' perspectives and expectations of care as an important contribution in and of itself (theirs is the underrepresented perspective in research), but also hope that sharing these insights supports primary health practitioners navigate the challenges they face in providing care. To that end, in tandem with health service providers, we have created a list of recommendations that are feasible within the confines of busy general practices.

These recommendations include prioritising staff education about refugees' previous experiences of healthcare overseas and the way these experiences shape expectations of care. To help achieve this, ‘Champions’ across ALL practice roles (GPs, nurses and receptionists) can undertake extra training and share information with the rest of the staff and be a consistent point of contact for staff who care for refugee patients and, where possible, refugee patients themselves. Practitioners should take time to explain the new health system to refugee patients (such as the role of GPs and why waitlists exist) and to discuss when practices of ‘best care’ may differ (such as different prescribing practices) and why. Some additional practical suggestions include: labelling ‘refugee background patients’ in general practice patient management systems, understanding the utmost importance of ‘the right’ interpreter to facilitate trust and accurate information gathering and remembering pharmacies may not have interpretation services (in New Zealand, they do not). Finally, while acknowledging the needs of refugee patients, practitioners should take care to avoid a ‘deficit lens’—refugees are survivors with skills and capacity. These recommendations are expanded upon, and more are provided, in a brief online resource [42] (also see two short videos [43, 44]).

### 4.1 Limitations

We acknowledge the limitations of our studies with small sample sizes based specifically in two regional sites across southern New Zealand. However, the care taken to address ethical and methodological complexities, and the relative scarcity of refugees' perspectives of healthcare, make this paper's input into refugee healthcare valuable. While healthcare delivery differs across New Zealand, our engagement with providers during the research, dissemination and development of general practice resources suggests that the experiences outlined here are common among refugees and the providers who work with them throughout the country. The findings are further applicable beyond New Zealand, for primary care systems that serve refugees used to hospital‐based care and easier access to specialists.

## 5 Conclusion

Former refugees report multiple challenges and high levels of dissatisfaction with healthcare in New Zealand. Refugees' experiences of healthcare in New Zealand are often shaped by prior experiences of healthcare before they even set foot in the country. These overseas experiences of healthcare are often dominated by direct and immediate access to specialists and faster and more frequent access to tests, procedures and medicines. There is a significant imbalance between expectations of healthcare, based on previous experience overseas, and the reality of healthcare provision in New Zealand's primary care system. We hope that by providing insight into refugees' previous healthcare experiences and how these shape their expectations of care, GPs will be better equipped to understand their refugee patients' perspectives—ultimately improving the care experience for both parties.

## Author Contributions

**Molly George:** conceptualisation, formal analysis, funding acquisition, investigation, methodology, project administration, supervision, writing – original draft. **Rebekah Kennedy:** formal analysis, investigation, validation, writing – review and editing. **Lauralie Richard:** conceptualisation, formal analysis, funding acquisition, investigation, methodology, validation, writing – review and editing. **Pauline Norris:** conceptualisation, formal analysis, funding acquisition, methodology, supervision, visualisation, validation, writing – original draft.

## Ethics Statement

Ethical approval was obtained from the University of Otago Human Ethics Committee (D18/052 and H20/154).

## Conflicts of Interest

The authors declare no conflicts of interest.

## Data Availability

The data that support the findings of this study are available from the corresponding author upon reasonable request.
